# Body‐identical estrogens in combined hormonal contraceptives: A safer path forward

**DOI:** 10.1002/ijgo.70393

**Published:** 2025-07-17

**Authors:** Nathalie Chabbert‐Buffet, Ivonne J. Diaz Yamal, Jonathan Douxfils, Jean‐Michel Foidart, Franca Fruzzetti, Kristina Gemzell‐Danielsson, Rossella E. Nappi, Yutaka Osuga, Philippe Descamps

**Affiliations:** ^1^ Department of Obstetrics, Gynecology and Reproductive Medicine Tenon Hospital AP‐HP Sorbonne University Paris France; ^2^ Universidades Unisanitas y Militar Nueva Granada Bogotá Colombia; ^3^ Department of Pharmacy, Namur Thrombosis and Hemostasis Center University of Namur Namur Belgium; ^4^ Department of Obstetrics and Gynecology University of Liège Liège Belgium; ^5^ Gynecological Endocrinology Unit San Rossore Clinical Center Pisa Italy; ^6^ Department of Women's and Children's Health Karolinska Institutet and Karolinska University Hospital Solna Stockholm Sweden; ^7^ Department of Clinical, Surgical, Diagnostic and Pediatric Sciences University of Pavia Pavia Italy; ^8^ Research Center for Reproductive Medicine, Gynecological Endocrinology and Menopause IRCCS Policlinico San Matteo Pavia Italy; ^9^ Teikyo Academic Research Center Teikyo University Tokyo Japan; ^10^ Department of Obstetrics and Gynecology University Hospital Angers Angers France

**Keywords:** body‐identical estrogens, estetrol, estradiol, FIGO, safety, venous thromboembolism, white paper

The estrogen component of combined hormonal contraceptives (CHCs) has long been a subject of clinical focus and debate. With growing awareness of estrogen‐related safety concerns, particularly regarding venous thromboembolism (VTE), there is increasing interest in reevaluating the type of estrogen used in CHCs.^1^ Robust clinical, biological, and epidemiological data now point to a significant safety advantage for natural/body‐identical estrogens, namely, estradiol (E2) and estetrol (E4), over synthetic ethinylestradiol (EE).

Given the growing interest in safer alternatives, prospective cohort studies have shown that E2‐based CHCs such as E2/NOMAC (E2 combined with nomegestrol acetate) and E2V/DNG (E2‐valerate combined with dienogest) are associated with significantly lower VTE risk than EE‐based formulations.^2^, ^3^ Similarly, biological studies by Gaussem et al. confirmed that E2 induces fewer changes in thrombin generation and activated protein C resistance than EE.^4^ A recent meta‐analysis involving over 560 000 women reported a significant (33%) reduction in VTE risk (odds ratio [OR] 0.67, 95% confidence interval [CI] 0.51–0.87) when EE was replaced by E2 in CHC formulations.^5^

Recent findings also support that E4, a fetal‐derived native estrogen, has a distinct mechanism of action with selective tissue activity and a mild hepatic impact.^6^ Controlled studies have demonstrated that either in combination with drospirenone (DRSP) or alone, E4 has no clinically significant effect on thrombin generation,^7^ and leads to a much lower effect on estrogen‐induced activated protein C resistance compared to EE‐based CHCs.^8^, ^9^ These findings are aligned with real‐world evidence from pharmacovigilance studies.^10^ The analysis demonstrated that combinations with body‐identical estrogens exhibited a significantly lower reporting rate of thrombotic events compared to EE‐LNG formulations. E2‐NOMAC and E2‐DNG showed comparable proportional reporting rates (PRR) to EE/LNG (0.44 [95% CI, 0.38–0.51] and 0.45 [95% CI, 0.41–0.49], respectively), while E4/DRSP had the lowest PRR (0.24 [95% CI, 0.17–0.33]). E4/DRSP exhibited a PRR similar to that observed for the progestogen‐only pill, DRSP 4 mg, supporting the neutral effect of E4 on coagulation and its consequent VTE risk (0.24 [95% CI, 0.19–0.29]) (Figure 1).^10^

**FIGURE 1:**
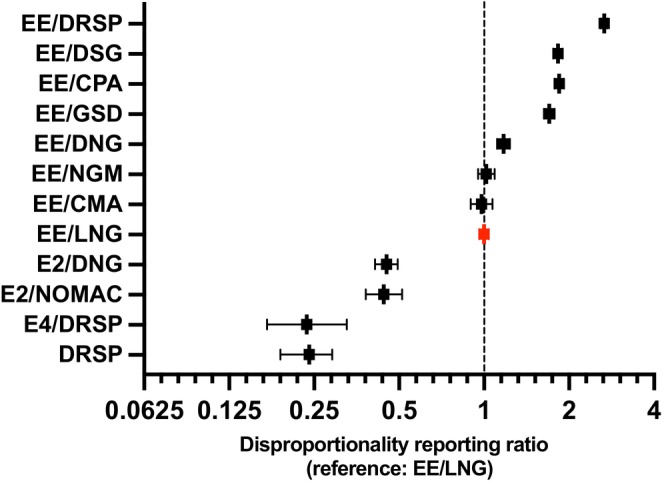
Disproportionality reporting ratios* and their 95% confidence intervals for each estroprogestative combination versus EE/LNG as the reference (adapted from Didembourg 2025).^10^ CMA, chlormadinone acetate; CPA, cyproterone acetate; DNG, dienogest; DRSP, drospirenone; DSG, desogestrel; GSD, gestodene; LNG, levonorgestrel; NGM, norgestimate; NOMAC, nomegestrol acetate. *The proportional reporting rate (PRR) is used to summarize the extent to which a particular adverse event is reported among individuals taking a specific drug, compared to the frequency at which the same adverse event is reported for individuals taking some other drugs in a specified class of drugs. The PRR is typically calculated using a surveillance database (EMA or FDA) in which reports of adverse events from a variety of drugs are recorded.

In response to this emerging evidence, an international group of experts, coordinated through the ESCONEC initiative (Expert Committee for Estrogens and Combined Oral Contraceptives), is developing a comprehensive white paper titled “The value of the estrogen component in combined hormonal contraceptives (CHCs).” The aim is to build a consensus on the clinical relevance of estrogen choice in CHCs, inform prescribers and policymakers, and support evidence‐based decision‐making around estrogen selection in contraceptive care.

This white paper draws on contributions from clinicians across all continents, ensuring a global perspective on the use of body‐identical estrogens in contraception. It aims to reflect the diversity of healthcare realities by integrating considerations such as patient preferences, health benefits, counseling needs, family planning goals, access, and affordability. Special attention is being given to cultural and racial/ethnic differences in contraceptive use, which are elements that are often underrepresented in the literature. The paper is structured into an executive summary and two main sections: one offering a clinically accessible rationale for selecting body‐identical estrogens in CHCs and the other providing detailed scientific analysis and comparative data on estrogen types.

Importantly, the white paper will be formally presented during a dedicated symposium at the upcoming International Federation of Gynecology and Obstetrics (FIGO) World Congress in Cape Town, South Africa (October 7, 2025). This platform will serve to disseminate the findings and stimulate broader discussions among key stakeholders in women's health.

We believe it is time for a paradigm shift in estrogen selection for CHCs. Body‐identical estrogens not only offer better safety but might also enhance user satisfaction and compliance through improved tolerability. We encourage healthcare providers, regulatory agencies, and professional organizations to closely follow the progress of this initiative and support its conclusions to achieve safer, more personalized contraceptive care.

## AUTHOR CONTRIBUTIONS

**Nathalie Chabbert‐Buffet:** Conceptualization, writing—original draft preparation, writing review and editing, supervision. **Jonathan Douxfils:** Writing—original draft preparation, writing review and editing. **Ivonne J. Diaz Yamal, Jean‐Michel Foidart, Franca Fruzzetti, Kristina Gemzell‐Danielsson, Rossella E. Nappi, Yutaka Osuga, and Philippe Descamps:** Writing review and editing. All authors gave final approval of the version to be submitted.

## FUNDING INFORMATION

This brief communication was supported by Gedeon Richter Plc, Hungary, through an unrestricted grant. The sponsor had no influence on the content or writing of the manuscript.

## CONFLICT OF INTEREST STATEMENT

NC‐B has served on Advisory Boards for BESINS, Exeltis, and Gedeon Richter. IDY chairs the Division of Sexual and Reproductive Health and Wellbeing at FIGO. JD is the director and founder of Qualiblood, a contract research organization that received funding from Mithra Pharmaceuticals and Fuji Pharma. He also reports personal fees from Daiichi Sankyo, Diagnostica Stago, Gedeon Richter, Portola, Roche, and Roche Diagnostics. JMF was a board member for Mithra, the company that initially developed the study product for contraception. FF has served as speaker and advisory board member for Theramex, Gedeon‐Richter, and Organon. KGD is an ad hoc advisory board member and speaker for Organon (MSD), Bayer, Exelgyn, Actavis, Gedeon Richter, Mithra, Exeltis, Ferring, Natural Cycles, Azanta, Gynuity, Obseva MedinCell, Cirqle, Addeira, and HRA‐Pharma. She is a member of the Population Councils International Committee for Contraception Research and director of a World Health Organization Collaborating Center for Research in Human Reproduction. REN has ongoing relationships with Abbott, Astellas, BayerHealthCare AG, Besins Healthcare, Biocodex, Exeltis, Fidia, Gedeon Richter, Merck & Co, Novo Nordisk, Organon & Co, Shionogi Limited, Theramex, Viatris, and Vichy Laboratories. She is the current president of the International Menopause Society (IMS). YO reports a medical expert contract from Fuji Pharma. PD reports financial compensation from Hologic for his activity as co‐chair of the ACCESS Consensus Group.

## Data Availability

Not applicable.
